# Drivers of community turnover differ between avian hemoparasite genera along a North American latitudinal gradient

**DOI:** 10.1002/ece3.6283

**Published:** 2020-06-09

**Authors:** Naima C. Starkloff, Jeremy J. Kirchman, Andrew W. Jones, Benjamin M. Winger, Yen‐Hua Huang, Paulo C. Pulgarín‐R, Wendy C. Turner

**Affiliations:** ^1^ Department of Biological Sciences University at Albany State University of New York Albany NY USA; ^2^ New York State Museum Albany NY USA; ^3^ Department of Ornithology Cleveland Museum of Natural History Cleveland OH USA; ^4^ Museum of Zoology and Department of Ecology and Evolutionary Biology University of Michigan Ann Arbor MI USA; ^5^ Laboratorio de Biología Evolutiva de Vertebrados Departamento de Ciencias Biológicas Universidad de Los Andes Bogotá Colombia; ^6^ Facultad de Ciencias & Biotecnología Universidad CES Medellin Colombia

**Keywords:** avian malaria, *Catharus*, community ecology, hemosporidia, latitudinal diversity gradient, *Leucocytozoon*

## Abstract

The latitudinal diversity gradient (LDG) is an established macroecological pattern, but is poorly studied in microbial organisms, particularly parasites. In this study, we tested whether latitude, elevation, and host species predicted patterns of prevalence, alpha diversity, and community turnover of hemosporidian parasites. We expected parasite diversity to decrease with latitude, alongside the diversity of their hosts and vectors. Similarly, we expected infection prevalence to decrease with latitude as vector abundances decrease. Lastly, we expected parasite community turnover to increase with latitudinal distance and to be higher between rather than within host species. We tested these hypotheses by screening blood and tissue samples of three closely related avian species in a clade of North American songbirds (Turdidae: *Catharus*, *n* = 466) across 17.5° of latitude. We used a nested PCR approach to identify parasites in hemosporidian genera that are transmitted by different dipteran vectors. Then, we implemented linear‐mixed effects and generalized dissimilarity models to evaluate the effects of latitude, elevation, and host species on parasite metrics. We found high diversity of hemosporidian parasites in *Catharus* thrushes (*n* = 44 lineages) but no evidence of latitudinal gradients in alpha diversity or prevalence. Parasites in the genus *Leucocytozoon* were most prevalent and lineage rich in this study system; however, there was limited turnover with latitude and host species. Contrastingly, *Plasmodium* parasites were less prevalent and diverse than *Leucocytozoon* parasites, yet communities turned over at a higher rate with latitude and host species. *Leucocytozoon* communities were skewed by the dominance of one or two highly prevalent lineages with broad latitudinal distributions. The few studies that evaluate the hemosporidian LDG do not find consistent patterns of prevalence and diversity, which makes it challenging to predict how they will respond to global climate change.

## Graphical abstract

Catharus thrushes are heavily infected by a diverse assemblage of hemosporidian parasites; however, these parasites did not follow a latitudinal diversity gradient or prevalence. Instead, we see high community turnover of *Plasmodium* communities with latitude and host species and moderate turnover of *Leucocytozoon* communities with latitude.

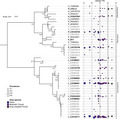

## 1 INTRODUCTION

One of the most universal patterns in ecology is the latitudinal diversity gradient (LDG), where communities become less species rich with increasing latitude (Hillebrand, 2004; Mannion, Upchurch, Benson, & Goswami, 2014). Numerous mechanisms have been proposed to explain this phenomenon (Preisser, 2019), including greater productivity in the tropics (Connell & Orias, 1964; Hawkins, Porter, & Felizola Diniz‐Filho, 2003), climatic stability at lower latitudes (Klopfer, 1959), time for diversification (Pianka, 1966), and diversification rate (Mittelbach et al., 2007). This pattern has been documented extensively in vertebrates (Davies & Buckley, 2011; Hawkins et al., 2003; Rabosky, Title, & Huang, 2015), plants (Davies, Savolainen, Chase, Moat, & Barraclough, 2004), and the majority of parasite‐transmitting insect vectors that have been studied (Foley, Rueda, & Wilkerson, 2007; Mullens, Gerry, Lysyk, & Schmidtmann, 2004). Given that hosts and vectors of parasites largely follow the LDG and previous studies have shown that parasite diversity correlates with host diversity (Hechinger & Lafferty, 2005; Watters, 1992), we expect the LDG to be ubiquitous in parasites (Bordes, Morand, Krasnov, & Poulin, 2010). However, parasite diversity patterns are not consistent with the LDG (Preisser, 2019).

Like all organisms, the niche of a parasite is substantially determined by its environmental tolerance (Holt, 2003; Nuismer & Kirkpatrick, 2003; Sexton, McIntyre, Angert, & Rice, 2009). A parasite inhabits one or more organisms throughout its lifecycle; thus, its niche and distribution are a consequence of a cascade of tolerances and interactions among parasite, vector, and/or host (Murdock, Foufopoulos, & Simon, 2013; Nuismer & Kirkpatrick, 2003). The range of a parasite species is also determined by its degree of host specificity as well as the composition of host communities (Clark et al., 2018). A host‐specific parasite will be limited by the presence of its co‐evolved host, whereas a generalist is able to parasitize many different host species and thereby occupy a greater geographic range (Hellgren, Pérez‐Tris, & Bensch, 2009). However, there may be trade‐offs to wide distributions, as specialists have been shown to achieve higher rates of infection than generalists within their co‐evolved host populations (Medeiros, Ellis, & Ricklefs, 2014). In addition, parasite ranges can be limited by the presence of geographic barriers to host or vector movement across the host species’ range, allowing susceptible hosts to remain uninfected (Brooks & Ferrao, 2005; Warburton, Kohler, & Vonhof, 2016).

Only a few studies present evidence of the LDG in parasites (Cumming, 2000; Guernier, Hochberg, & Guégan, 2004; Nunn, Altizer, Sechrest, & Cunningham, 2005; Rohde, 1978). More often, latitude does not predict diversity patterns (Clark, 2018; Guilhaumon, Krasnov, Poulin, Shenbrot, & Mouillot, 2012; Illera, Fernández‐Álvarez, Hernández‐Flores, & Foronda, 2015; Kamiya, O’Dwyer, Nakagawa, & Poulin, 2014; Merino et al., 2008; Poulin, 1995; Thieltges, Ferguson, Jones, Noble, & Poulin, 2009) or parasite diversity increases with latitude resulting in a reverse LDG (Blaylock, Margolis, & Holmes, 1998; Calvete, Estrada, Lucientes, Estrada, & Telletxea, 2003; Choudhury & Dick, 2000; Cuevas et al., 2020; Fecchio et al., 2019; Krasnov, Shenbrot, Khokhlova, & Degen, 2004; Linardi & Krasnov, 2013; Lindenfors et al., 2007). There is no consistency of latitudinal diversity patterns even within some taxa; for example, flea diversity increases with latitude in rodents in Brazil (Krasnov et al., 2004) but does not vary with latitude in small mammals globally (Linardi & Krasnov, 2013). Similarly, avian blood parasite diversity does not vary significantly with latitude in a Chilean study (Merino et al., 2008) but increases with latitude in two studies with larger geographic scales (Cuevas et al., 2020; Fecchio et al., 2019). Finding the appropriate taxonomic scale (of both host and parasite) at which to test for the typical and reverse LDG of parasites remains a challenging task (Preisser, 2019).

While alpha diversity is typically quantified in LDGs, community turnover (dissimilarity in community composition between sites, or beta diversity) provides an evaluation of compositional variation. Parasite community turnover is typically predicted by geographic distance (Nekola & White, 1999); however, it can also vary with ecological factors such as land use (Warburton et al., 2016) and elevation (Williamson et al., 2019) and host factors such as host phylogeny and connectivity (Clark et al., 2018). Parasite communities are likely to turnover with latitudinal distance, and at higher rates between than within host species.

In addition to surveying parasite diversity, parasite prevalence (the proportion of individuals infected in a site) is a central metric to disease ecology (Krasnov & Poulin, 2010). Prevalence indicates the relative success of a parasite group in infecting a particular host community or population (Garcia‐Langoria, Marzal, De Lope, & Garamszegi, 2019). The prevalence of vector‐transmitted parasites is likely to decrease with latitude as temperature decreases with latitude, resulting in slower rates of development and a reduction in the number of generations of insect vectors in a single season (Gage, Burkot, Eisen, & Hayes, 2008). This should lead to a decrease in vector abundance with latitude (Hesson, Östman, Schäfer, & Lundström, 2011) and, ultimately, in the infection prevalence of parasites. For example, Merino et al. (2008) found the typical and reverse latitudinal gradients of prevalence of different genera of hemosporidian parasites despite not finding support for similar latitudinal gradients in diversity.

We investigated the latitudinal variation in parasite diversity (alpha and beta) and infection prevalence in a model parasite system (Order Haemosporidia) infecting a clade of three closely related avian host species. These parasites cause acute infections that vary in the severity of symptoms, but generally result in some level of anemia, enlargement of liver and spleen, and infection of phagocytes (Valkiunas, 2005). Chronic infections can have long‐term effects on lifespan, offspring number, and offspring quality of their avian hosts (Asghar et al., 2015). Avian hemosporidian infections are caused by parasites in three genera (Valkiunas, 2005): *Plasmodium* (transmitted by mosquitoes in the family Culicidae and traditionally referred to as “avian malaria” parasites), *Haemoproteus* (subgenus *Parahaemoproteus*, transmitted by midges in the family Ceratopognidae), and *Leucocytozoon* (transmitted by blackflies, family Simuliidae). Hemosporidians vary greatly in degree of specialization on hosts (Valkiunas, 2005) and have thermal and rainfall tolerances which limit their geographic distribution (Jones, Cheviron, & Carling, 2013; LaPointe, Atkinson, & Samuel, 2012), which may lead to variation in parasite assemblages with latitude. We generally expect hemosporidian parasites to follow the LDG along with their avian hosts and dipteran vectors. However, *Leucocytozoon* prevalence and alpha diversity should correlate positively with latitude (reverse LDG) as they are transmitted by blackflies whose abundance (McCreadie & Adler, 2014) and species richness (McCreadie, Williams, Stutsman, Finn, & Adler, 2017) are also positively correlated with latitude. As a result, *Leucocytozoon* parasites persist at high latitudes and elevations (Haas, Lukán, Kisková, & Hrehová, 2012; Oakgrove et al., 2014). Very little is known about how host range limits determine the distribution of hemosporidians in wildlife populations. However, studies of diversity across large geographic scales have become more accessible and robust with the shift of identification from microscopy to molecular markers (Bensch, Hellgren, & Pérez‐Tris, 2009; Hellgren, Waldenström, & Bensch, 2004).

**FIGURE 1:**
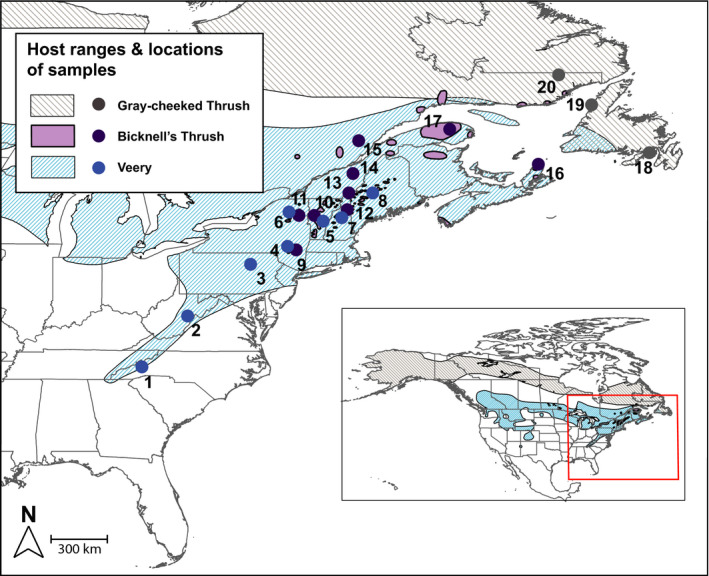
The localities of 20 sampling sites (numbers correspond to sites in Table 1) and the breeding ranges of the three host species (Birdlife International & Nature Serve, 2014). The inset indicates the continental breeding ranges, and the red box within it shows the location of the 20 sampling sites. Only one species was sampled at each site, as they do not co‐occur, segregated based on elevation and latitude

We use mitochondrial DNA sequence data to document hemosporidian parasites infecting a clade of three migratory *Catharus* thrush species sampled on their breeding grounds across a gradient of 17.5° of latitude in eastern North America. *Catharus* thrushes are well‐studied phylogenetically (Everson et al., 2019; FitzGerald et al., 2020; Voelker, Bowie, & Klicka, 2013) and ecologically (Able & Noon, 1976; FitzGerald, 2017; Noon, 1981). The sister species Bicknell's Thrush (*C. bicknelli*) and Gray‐cheeked Thrush (*C. minimus*) breed in boreal forests dominated by fir (*Abies*) and spruce (*Picea*) forests (FitzGerald, 2017) and are latitudinal replacements of one another (Figure 1). Their closest relative, the Veery (*C. fuscescens*), breeds in temperate deciduous forest and is an altitudinal replacement of *C. bicknelli* (Noon, 1981). All three species are morphologically similar and have similar diets and nesting ecologies, yet they rarely co‐occur, and hybridization events are extremely rare (FitzGerald, 2017; Martinsen, McFarland, & Rimmer, 2018). Screening for hemosporidian parasites hosted in these three species across a large geographic gradient allows for the evaluation of latitudinal and elevational effects on diversity and prevalence and for estimating the contribution of host specificity. In addition, *Catharus* thrushes have high hemosporidian prevalence relative to other genera of birds (Greiner, Bennett, White, & Coombs, 1975), such that analysis of parasite community structure will be based on larger samples than would be possible from a similar sample of different avian hosts. Lastly, our focus on intensively sampled and closely related species enables us to document host specificity with greater precision than studies that evaluate a single host species or a community of birds with small sample sizes of each individual species.

Our central goals are to (i) document the alpha and beta diversity and infection prevalence of three genera of hemosporidian parasites infecting *Catharus* thrushes and (ii) evaluate the role of latitude, elevation and host species on variation in these three parasite metrics.

## 2 MATERIALS AND METHODS

### 2.1 Field sampling

We screened blood and tissue samples from adult Veeries (*n* = 190), Bicknell's Thrushes (*n* = 207), and Gray‐cheeked Thrushes (*n* = 68) in 20 sites across eastern North America (Figure 1, Table 1) during the breeding seasons of 1993–2018 (late May to early July). Blood samples were obtained using targeted mist netting and brachial venipuncture from banded and released birds by researchers from the Vermont Center for Ecostudies, Canada Wildlife Society, and New York State Museum (NYSM). Tissue samples were frozen prior to study and are associated with voucher specimens archived at the Cleveland Museum of Natural History, American Museum of Natural History, and NYSM. Studies have found no significant difference in the detectability of hemosporidian parasites in blood samples as compared to a number of different tissue types (Pulgarín‐R et al., 2018; Ramey, Fleskes, Schmutz, & Yabsley, 2013; Svensson‐Coelho et al., 2016). Combining existing samples with new field collections allowed us to address questions over a larger geographic scale than would otherwise be possible. While the variation in the years of sample collection is a potential limitation of our study, several studies demonstrated that hemosporidian prevalence patterns remain relatively constant over time (Lachish et al., 2013; Pagenkopp, Klicka, Durrant, Garvin, & Fleischer, 2008; Pulgarín‐R et al., 2018). This pattern is likely due to the chronic nature of these infections (Valkiunas, 2005). We include timespan of sampling at each study site as a random variable in our models to account for its effects on the prevalence or diversity of hemosporidian parasites (see Analyses).

**TABLE 1:** Host species, latitude (°North), elevation (meters above sea level), sampling, raw lineage richness (α diversity), and raw infection prevalence (proportion of sample size infected) at 20 sites

Site #	Host species	Site name	Mean latitude	Mean elevation	Sample size	Number of lineages				Infection prevalence		
						Total	*Haem.*	*Plasm.*	*Leuc.*	*Haem.*	*Plasm.*	*Leuc.*
1	Veery	Appalachian Mountains, North Carolina	35.51	1,306	19	6	0	3	3	0.00	0.42	0.89
2	Veery	Allegheny Mountains, West Virginia	38.67	1,132	20	9	2	3	4	0.10	0.55	0.90
3	Veery	Pocono Mountains, Pennsylvania	41.38	469	19	9	1	4	4	0.05	0.32	0.84
4	Veery	Catskill Mountains, New York	42.27	522	45	10	2	3	5	0.04	0.09	0.73
5	Veery	Green Mountains, Vermont	43.95	458	28	12	0	5	7	0.00	0.36	0.89
6	Veery	Adirondack Mountains, New York	44.13	472	21	10	0	3	7	0.00	0.48	0.95
7	Veery	White Mountains, New Hampshire/Maine	44.07	435	20	7	0	1	6	0.00	0.10	1.00
8	Veery	Weyerhaeuser Timber, Maine	45.53	360	18	7	0	2	5	0.00	0.17	1.00
9	Bicknell's Thrush	Catskill Mountains, New York	42.16	1,168	41	7	0	1	6	0.00	0.07	0.73
10	Bicknell's Thrush	Green Mountains, Vermont	44.13	1,141	27	8	0	3	5	0.00	0.19	0.81
11	Bicknell's Thrush	Adirondack Mountains, New York	44.18	1,221	25	9	0	2	7	0.00	0.16	0.88
12	Bicknell's Thrush	White Mountains, New Hampshire/ Maine	44.52	1,412	20	7	1	1	5	0.05	0.10	0.80
13	Bicknell's Thrush	Mont Gosford, Quebec	45.30	1,127	20	12	2	2	8	0.20	0.25	0.95
14	Bicknell's Thrush	Massif du Sud, Quebec	46.61	836	19	11	2	3	6	0.16	0.32	1.00
15	Bicknell's Thrush	Lac Poulin, Quebec	47.91	923	20	6	1	1	4	0.10	0.30	0.90
16	Bicknell's Thrush	Northern Nova Scotia	47.06	32	15	5	0	2	3	0.00	0.33	1.00
17	Bicknell's Thrush	Gaspésie, Quebec	48.86	615	20	7	0	1	6	0.00	0.05	0.90
18	Gray‐cheeked Thrush	Southern Newfoundland	47.32	47	29	9	2	3	4	0.07	0.28	0.41
19	Gray‐cheeked Thrush	Northern Newfoundland	50.35	340	15	7	2	1	4	0.47	0.13	0.60
20	Gray‐cheeked Thrush	Labrador	52.53	331	24	12	3	3	6	0.25	0.17	0.92

### 2.2 Parasite screening

Whole genomic DNA was extracted using a DNeasy Blood and Tissue Extraction Kit (QIAGEN, Valencia, CA). We used a nested PCR approach that targeted a 479 bp fragment of the parasite mitochondrial gene cytochrome *b* (cyt‐*b*) (Hellgren et al., 2004). The first step targets and amplifies cyt‐*b* from all three hemosporidian genera (primers HaemNFI and HaemNR3). The second round of PCR targets either of the two closely related groups *Haemoproteus* and *Plasmodium* (primers HaemF and HaemR2) or *Leucocytozoon* (primers HaemFL and HaemR2L). Negative PCRs (indicated by the lack of band on 1.5% agarose gels) were repeated twice (for a total of three times) as false negatives are common (24% of *Leucocytozoon* and 15% *Haemoproteus* or *Plasmodium* infections were detected after the first screening). Positive amplification products were cleaned using Exosap (ExoSAP‐IT; Amersham Biosciences, Arlington Heights, IL) and Sanger‐sequenced on an ABI3700. Sequences were aligned and edited using Sequencher (version 5.4.6; Gene Codes Corporation, Ann Arbor, MI USA), and lineages identified by comparison to known sequences from MalAvi (Bensch et al., 2009) and GenBank (Clark, Karsch‐Mizrachi, Lipman, Ostell, & Sayers, 2015).

Parasites with even a single, unambiguous nucleotide difference are assigned to different, potentially reproductively isolated lineages (Bensch, Pérez‐Tris, Waldenströum, & Hellgren, 2004; Perez‐Tris & Bensch, 2005). Sequences not matching known lineages in MalAvi or GenBank are considered novel lineages and were deposited in MalAvi. As the nested PCR protocol does not reliably detect hemosporidian coinfections of the same genus, we do not address coinfection in this paper (Soares, Latta, & Ricklefs, 2019). We evaluated the phylogenetic relationship among lineages based on mitochondrial DNA sequences using maximum likelihood (ML) with 1,000 replicate bootstrap support in MEGA7 (Kumar, Stecher, & Tamura, 2016). We determined the best model for our data (GTR + G + I substitution model) using the “modelTest” function in the R package “phangorn” (Schliep, 2011). The ML tree was rooted at the *Leucocytozoon* clade (Marroquin‐Flores et al., 2017), a relationship established by Borner et al. (2016) using multiple loci and taxa.

### 2.3 Analyses

We used linear mixed‐effects models (LMM; “lmer” function in the R package “lme4”) of varying combinations of predictive variables (see Appendix S1) for the alpha diversity and prevalence of each hemosporidian genus across the 20 sites and assessed model fit using ∆AICc (Burnham & Anderson, 2004). Latitude (mean of samples at each site), elevation (mean of samples at each site), and host species were included as potential fixed effects. To account for variation in the number of years across which birds were sampled in each site, we included a random effect where each site was assigned to one of three sampling categories: short (<5 years), mid (5–15 years), and long (>15 years). Prevalence was arcsine transformed to normalize the data (Ricklefs et al., 2005). Alpha diversity at each site was calculated based on the Shannon Diversity Index following several hemosporidian studies (Ferraguti et al., 2018; Jones et al., 2018). We used the R package “iNext” (Hseih, Ma, & Chao, 2016) which incorporates rarefaction to extrapolate diversity metrics, allowing for variation in sample size across sites. The Shannon index was calculated across sites for each parasite genus with 1,000 bootstrap and 95% confidence intervals.

To evaluate the contribution of predictors (latitude, elevation, and host species) in determining parasite community turnover within each hemosporidian genus, we used a generalized dissimilarity model (GDM; “gdm” function in the R package “gdm”). This function transforms predictor variables into a series of I‐spline functions and fits models using maximum‐likelihood estimation (Ferrier, Manion, Elith, & Richardson, 2007). These fitted I‐splines provide a rate of parasite community turnover relative to variation in a particular environmental or geographic gradient across sites (Fitzpatrick et al., 2013). We used the “gdm.varImp” function with 1,000 permutations to identify (a) the percentage deviance explained by the full model (containing all predictors) and (b) the variable importance of each predictor when removed from the full model. Biological distance among sites (parasite community dissimilarity) was calculated within the “formatsitepair” function using the “chao” index**,** which accounts for the variation in parasite abundance across sites (Oksanen et al., 2013). GDMs allow for the inclusion of a categorical variable, if coded as 0 and 1 using bioformat = 4 (Ferrier et al., 2007). To assess the role of host species on community turnover, each site comparison was assigned 0 or 1; that is, site comparisons were assigned a 0 when comparing two sites occupied by the same species (conspecific; Bicknell's‐Bicknell's or Veery‐Veery) or a 1 if one site was occupied by the Bicknell's Thrush and the other by the Veery (heterospecific). Sites occupied by the Gray‐cheeked Thrush were removed from this analysis as the number of conspecific site comparisons was too low (*n* = 3). For all analyses in R, we used version 3.5.2 (R Core Team, 2018).

## 3 RESULTS

**FIGURE 2:**
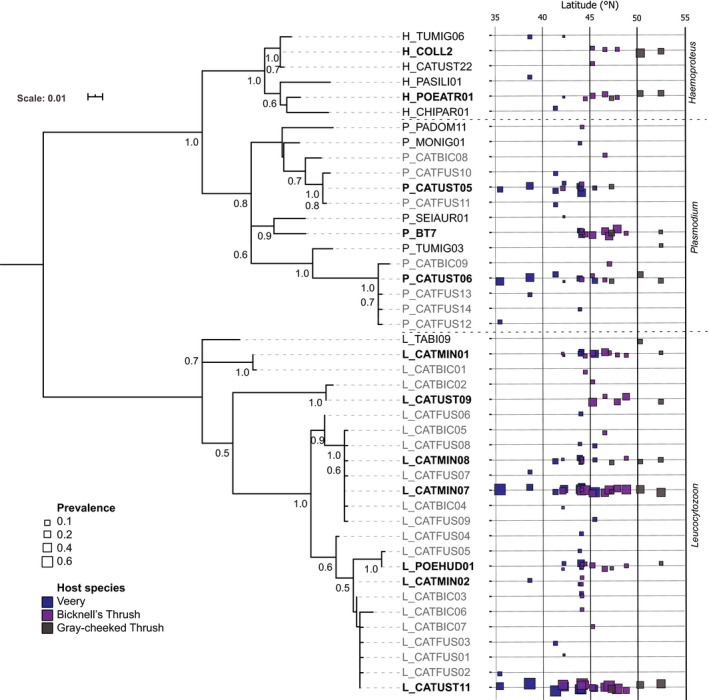
Maximum‐likelihood phylogenetic tree of hemosporidian parasite lineages in three genera *Haemoproteus*, *Plasmodium*, and *Leucocytozoon* based on cytochrome‐*b* DNA sequences. Branch lengths were determined by nucleotide changes (scale top left). Bootstrapping to assess node support is displayed for nodes with support between 0.5 and 1. Bolded lineages are common (infecting more than five individuals and found at more than two sites) and grayed lineages were not previously named or documented. Infection prevalence (proportion infected) of each lineage in sites across the latitudinal gradient is indicated by the size of the boxes and colors of boxes represent the host species present in each site

In our sample of 465 thrushes in three species, we found 44 lineages of hemosporidian parasites infecting 407 individuals (Figure 2). *Leucocytozoon* infections were the most prevalent (mean 86% across sites, Table 1) and diverse (24 lineages total, Figure 2) across all three host species, followed by *Plasmodium* (24% infected, 14 lineages) and *Haemoproteus* (7% infected, 6 lineages). Twelve lineages were relatively common (occurring in at least five individuals and found in more than two sites) and infected all three host species in the majority of cases (Figure 2). Six of these common hemosporidian lineages were found only north of 42°N, while three were only found south of 47°N (Figure 2). The remaining three common lineages were found throughout the sampling gradient. Across the latitudinal gradient, we found a large number of lineages that were amplified in just one or two individuals, many of which have not been documented in previous studies (17 *Leucocytozoon* and seven *Plasmodium* lineages). Most of these rare lineages differed by just 1–4 base pairs from commonly occurring parasite lineages (Figure 2).

**FIGURE 3:**
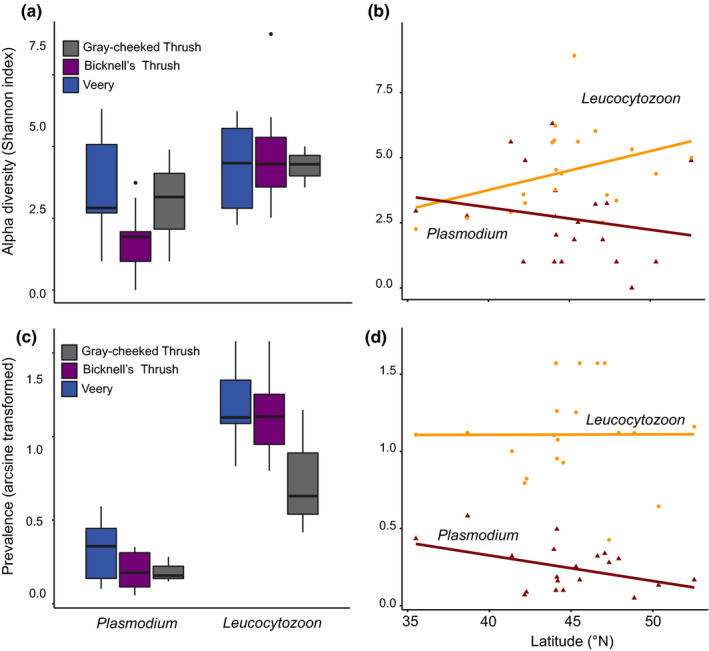
Mean hemosporidian alpha diversity (a) and prevalence by parasite genus (c) for each host species. Latitudinal variation in alpha diversity (b) and prevalence (d) by parasite genus for each host species. Alpha diversity is measured using the Shannon Diversity Index and prevalence was arcsine transformed. Only the variation in *Plasmodium* alpha diversity is supported by linear mixed‐effects models (Appendix S1)

The LMM model for *Plasmodium* alpha diversity suggests that the Bicknell's Thrush had lower lineage diversity than its congeners (Figure 3a, Appendix S1). There are a number of additional apparent trends: Gray‐cheeked Thrush had lower prevalence of *Leucocytozoon* than its congeners (Figure 3a), and *Plasmodium* prevalence and diversity decreased with latitude (Figure 3b,d), while *Leucocytozoon* alpha diversity increased with latitude (Figure 3b). However, with the exception of the *Plasmodium* alpha diversity model, the best mixed‐effects models were the intercept only models (Appendix S1). Latitude and elevation were not good predictors of alpha diversity or prevalence of either *Plasmodium* or *Leucocytozoon* parasites. We were not able to model *Haemoproteus* prevalence or diversity as parasites in this genus were absent from half of the sites.

**FIGURE 4:**
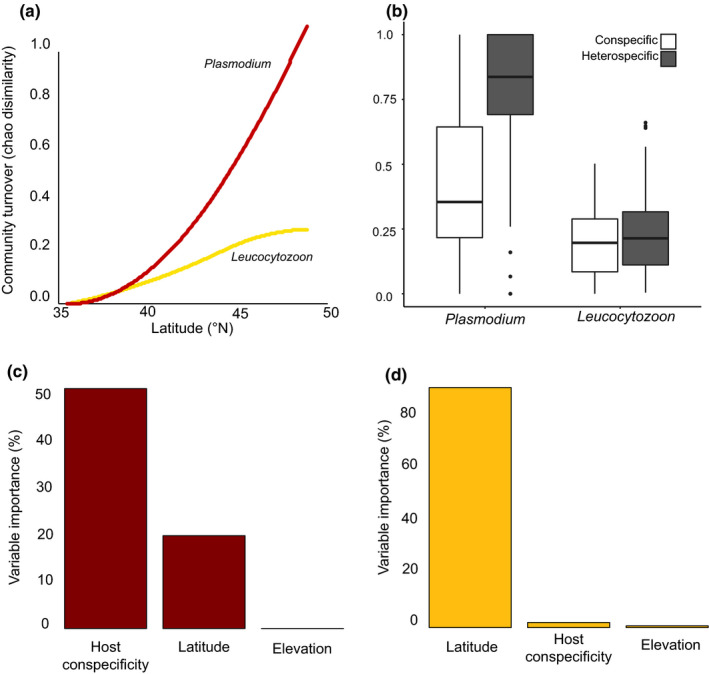
Fitted I‐splines of generalized dissimilarity models (partial regression fits) for community turnover with latitudinal distances in *Plasmodium* and *Leucocytozoon* (a). Maximum spline height provides an indication of the total turnover associated with latitude in each model. Mean turnover of *Plasmodium* and *Leucocytozoon* parasite communities between sites with the same host species (conspecific) or with different host species (heterospecific) (b). Variable importance (%) of each predictor in determining the turnover in *Plasmodium* (c) and *Leucocytozoon* (d) parasite communities. Variable importance is defined by the change in deviance explained when each predictor is permuted from the full model. The Gray‐cheeked Thrush was not included in this analysis

The GDM model for *Plasmodium* turnover explained a higher proportion of variance (32.09%) than for the *Leucocytozoon* model (12.52%). *Leucocytozoon* communities turn over at a lower rate than *Plasmodium* communities across the latitudinal gradient (Figure 4a). Host conspecificity was the best predictor of *Plasmodium* turnover (variable importance of 52.05%, Figure 4c), followed by latitude (variable importance of 21.15%, Figure 4c). *Plasmodium* communities turned over at a higher rate between sites with different host species (heterospecific) than with the same host species (conspecific), a pattern not seen among *Leucocytozoon* communities (Figure 4b). *Leucocytozoon* turnover was best described by just latitude (variable importance of 91.92%, Figure 4d). Elevation was not a good predictor of turnover of either parasite genus (Figure 4c,d).

## 4 DISCUSSION

While the LDG is an established ecological pattern for macrobiota, this pattern is not well understood for microbiota, especially hemosporidian parasites. Clark (2018) and Merino et al. (2008) found that latitude did not predict the diversity of any of the three avian hemosporidian genera, while Fecchio et al. (2019) and Cuevas et al. (2020) document an inverse LDG in *Leucocytozoon* parasites. Latitudinal increases in *Leucocytozoon* prevalence and diversity are thought to be related to their vector biology (Fecchio et al., 2019), as *Leucocytozoon* parasites are transmitted by blackflies, which also increase in abundance and species richness with increasing latitude (McCreadie & Adler, 2014; McCreadie et al., 2017). In contrast, mosquitoes follow the more common temperature–metabolism relationship: Mosquitoes decrease in their development rate, abundance, and vectoral capacity with lower temperatures (Gage et al., 2008; Lindsay & Birley, 1996). Thus, we expected *Plasmodium* parasites to follow the LDG. Our study shows trends of increasing *Leucocytozoon* alpha diversity and decreasing *Plasmodium* alpha diversity and prevalence with increasing latitude (Figure 3); however, these patterns were not supported by the LMMs (Appendix S1).

We did not find evidence for the classic or reverse LDG in either hemosporidian genus, however, we found that parasite community composition was increasingly different with latitudinal distance. *Leucocytozoon* communities turn over at a lower rate than *Plasmodium* communities (Figure 4a) as they tended to be dominated by one or two broadly distributed lineages (L_CATUST11 and L_CATMIN07), each reaching up to ~65% infection prevalence in certain sites (Figure 2). The majority of the remaining common *Leucocytozoon* lineages also occurred broadly but were absent at lower latitudes (Figure 2), which is likely driving the latitudinal turnover of *Leucocytozoon* communities (Figure 4a) and the apparent alpha diversity trend (Figure 3b). Contrastingly, *Plasmodium* communities had only a single parasite lineage (P_CATUST06) with a broad latitudinal range but did not exceed an infection prevalence of 31% (Figure 2). The two remaining common *Plasmodium* lineages seem to replace each other latitudinally: P_BT7 occurs only at higher latitudes and P_CATUST05 only at lower latitudes (Figure 2). Understanding the mechanisms that mediate the boundaries of lineages with restricted latitudinal ranges requires the identification of thermal thresholds necessary to complete their lifecycles and the host specificity of their dipteran vector species (Gage et al., 2008).

In addition to high rates of turnover of *Plasmodium* communities with latitudinal distance (Figure 4a), *Plasmodium* communities turn over at a higher rate between sites occupied by different host species (heterospecific) than between sites occupied by the same host species (conspecific), a pattern not seen in *Leucocytozoon* communities (Figure 4b). The breeding distributions of the Veery and Bicknell's Thrush do not overlap; the Veery is typically found at low elevations and the Bicknell's Thrush at high elevations (Able & Noon, 1976; Noon, 1981). Williamson et al. (2019) documented that hemosporidian communities experience high turnover with elevation within a single host species due to variation in climate and avian community composition across several elevational gradients. Whereas *Leucocytozoon* parasites thrive in cold climates (Fecchio et al., 2019; Oakgrove et al., 2014), *Plasmodium* parasites tend to decrease in prevalence with decreasing temperature (Zamora‐Vilchis, Williams, & Johnson, 2012). Consequently, there is likely elevational dropout of certain *Plasmodium* lineages as is suggested by the lower alpha diversity in the Bicknell's Thrush than the Veery (Figure 3a) and high community turnover between the two host species (Figure 4b). Our latitudinal study framework is not appropriate to test this elevation hypothesis directly as we cannot disentangle these two factors with the available data: Both the Veery and Bicknell's Thrush occur at lower elevations as latitude increases (Table 1). A survey across multiple elevational transects at smaller geographic scales would shed light on this hypothesis. If elevation is indeed an important determinant of parasite diversity, the decreasing elevational range of the Veery and Bicknell's Thrush with increasing latitude may account for the lack of strong support we found for hemosporidian LDGs. Deciduous‐coniferous ecotones occur at lower elevations with increasing latitude in the Appalachian highlands (Cogbill & White, 1991), and *Catharus* thrushes seem to modulate the climatic gradient across the latitudinal span of this study by residing at lower elevations farther North. Cuevas et al. (2020) documented a reverse LDG in *Leucocytozoon* parasites across 16 latitudinal degrees of South America using a single bird species as, unlike *Catharus* thrushes, the Thorn‐tailed Rayadito (*Aphrastura spinicauda*) is able to occupy four different habitats across the latitudinal gradient.

The observed turnover in *Plasmodium* communities may instead reflect the allopatric wintering grounds of these host species. The Bicknell's Thrush winters in the Greater Antilles and the Veery winters in South America (Clement, 2000), which is likely to result in an exposure to different parasite and vector communities. Migratory host species can become infected on wintering grounds or at migratory stopover sites (Altizer, Bartel, & Han, 2011; Waldenström, Bensch, Kiboi, Hasselquist, & Ottosson, 2002), which could also explain why we did not find evidence for the classic or reverse LDG of hemosporidian parasites. For example, some *Plasmodium* lineages (P_CATUST05, P_CATUST06 and P_PADOM01) in our study were also documented in South American resident bird species at a migratory stopover site of the Gray‐cheeked Thrush (Pulgarín‐R et al., 2018). However, the former two lineages and P_BT7 have also been documented in juvenile birds on the breeding grounds before their first migration (Cozzarolo, Jenkins, Toews, Brelsford, & Christe, 2018; Pulgarín‐R et al., 2018, Starkloff, unpubl.), suggesting that *Plasmodium* lineages may be broadly transmitted. More rigorous vector‐based studies (Bernotienė, Žiegytė, Vaitkutė, & Valkiūnas, 2019) are required to confirm local completion of hemosporidian life cycles on the wintering grounds, breeding grounds, and stopover sites. Soares et al. (2019) argue that winter transmission is likely the exception rather than the rule as migratory birds and the Neotropical residents that occupy their wintering grounds are typically infected by distinct hemosporidian parasite communities. Some hypotheses they suggest that warrant further investigation include temporal mismatches between peak vector abundance and the presence of migrants or parasite adaptation to local hosts preventing infection of migrants.

While *Plasmodium* parasites may have been transmitted throughout the range of their hosts, *Leucocytozoon* parasites are likely transmitted exclusively on the breeding grounds as their vectors are most abundant and diverse at higher latitudes (McCreadie & Adler, 2014; McCreadie et al., 2017). Consequently, *Leucocytozoon* parasites are rarely found in tropical latitudes (Fecchio et al., 2019) where *Catharus* thrushes winter (Clement, 2000). Additionally, *Leucocytozoon* community turnover is equivalent between and within host species (Figure 4b) despite the Veery and Bicknell's Thrush wintering allopatrically (Clement, 2000). *Leucocytozoon* parasites tend to specialize on a single host species or genus (Galen, Nunes, Sweet, & Perkins, 2018), suggesting that spillover from unrelated neotropical birds is unlikely. Lastly, the two most prevalent and ubiquitous *Leucocytozoon* parasite lineages (L_CATUST11 and L_CATMIN07) have been documented in juvenile thrushes across the continent before their first migration (Dodge, Guers, Sekercioğlu, & Sehgal, 2013, Pulgarín‐R et al., 2018, Starkloff unpubl.), confirming that they are transmitted in the breeding grounds.

We found latitudinal turnover in parasite communities of two hemosporidian genera, despite a lack of support for latitudinal gradients of parasite prevalence or alpha diversity. We also found that *Plasmodium* communities turned over at a higher rate than *Leucocytozoon* communities between than within host species and the Bicknell's Thrush had lower *Plasmodium* alpha diversity than its congeners. These patterns are likely due to variation in ecological conditions associated with allopatric wintering and breeding distribution. Our study is one of few latitudinal investigations of hemosporidian diversity and prevalence (Clark, 2018; Cuevas et al., 2020; Fecchio et al., 2019; Merino et al., 2008), and the inconsistent findings among studies suggest that there is still much to learn about parasite macroecology. Several regions of the world, such as southern South America, Canada, Asia, Central Africa, and Australia, are underrepresented in the MalAvi database (Bensch et al., 2009; Clark, 2018), limiting global conclusions on the LDG of hemosporidian parasites. The lack of a baseline regarding the biogeography and diversity of parasite groups makes it challenging to understand how they will respond to global change (Carson et al., 2020).

## CONFLICTS OF INTEREST

The authors confirm that there are no known conflict of interests and that the funders of this research had no input into this manuscript.

## AUTHOR CONTRIBUTIONS

NCS, WCT, and JJK conceived the ideas and designed methodology; NCS, AWJ, and BMW conducted the field work; NCS and PP did the laboratory work; NCS and YH conducted statistical analyses; NCS, WCT, and JJK wrote this manuscript; BMW, AWJ, YH, and PP provided comments on manuscript.

### Open Research Badges

This article has earned an Open Data Badge for making publicly available the digitally‐shareable data necessary to reproduce the reported results. The data is available at Appendix S2.

## Supporting information

Appendix S1

Appendix S2

## Data Availability

Sequences associated with novel lineages found in this study have been deposited in MalAvi (Bensch et al., 2009). Raw data associated with each sample (locality and infection data) are in Appendix S2.

## APPENDIX 1

Comparison of linear mixed‐effects models predicting the prevalence and alpha diversity of *Leucocytozoon* and *Plasmodium* parasites in three closely related species of *Catharus* thrushes (*n* = 465) across the 20 sites. We did not model *Haemoproteus* prevalence or diversity as infections were rare (mean 7% infected). Models are ranked in ascending order of ∆AICc, relative to the model with the lowest AICc for that parasite genus. The lowest AICc is indicative of the best model in the set. K indicates the number of parameters in each model. W represents the Akaike weight which is a measure of probability of the model being the best in the set. All models included sampling timespan as random effects, including the intercept only model. Prevalence was arcsine‐transformed and alpha diversity was quantified by extrapolating the Shannon Diversity Index at each site.

**Table:**

Dependent variable	Variables included	AICc	ΔAICc	K	W
*Leucocytozoon* prevalence	intercept only	19.98	0.00	3	0.899
	host species	24.86	4.88	5	0.078
	host species + latitude	28.32	8.35	6	0.014
	latitude	29.27	9.29	4	0.009
	elevation	38.60	18.62	4	0.000
	host species + elevation	41.28	21.30	6	0.000
	host species *latitude	44.58	24.61	8	0.000
	latitude + elevation	47.96	27.99	5	0.000
	host species + latitude + elevation	48.21	28.24	7	0.000
	host species*latitude + elevation	65.27	45.30	9	0.000
*Plasmodium* prevalence	Intercept only	−8.26	0.00	3	0.934
	latitude	−2.72	5.54	4	0.058
	host species	1.29	9.55	5	0.008
	host species+ latitude	10.66	18.92	6	0.000
	elevation	11.83	20.09	4	0.000
	latitude + elevation	17.38	25.64	5	0.000
	host species + elevation	21.85	30.11	6	0.000
	host species*latitude	29.38	37.63	8	0.000
	host species + latitude + elevation	31.65	39.90	7	0.000
	host species*latitude + elevation	52.01	60.27	9	0.000
*Leucocytozoon* alpha diversity	Intercept only	80.09	0.00	3	0.735
	host species	83.97	3.87	5	0.106
	latitude	83.97	3.88	4	0.106
	host species + latitude	85.42	5.33	6	0.051
	host species*latitude	92.95	12.86	8	0.001
	elevation	95.69	15.60	4	0.000
	host species + latitude + elevation	97.81	17.72	7	0.000
	latitude + elevation	99.28	19.19	5	0.000
	host species + elevation	100.06	19.97	6	0.000
	host species*latitude + elevation	108.39	28.30	9	0.000
*Plasmodium* alpha diversity	Host species	83.02	0.00	5	0.701
	intercept only	85.09	2.07	3	0.249
	host species + latitude	89.29	6.26	6	0.031
	latitude	90.28	7.25	4	0.019
	host species + elevation	99.04	16.02	6	0.000
	elevation	99.19	16.16	4	0.000
	host species*latitude	99.29	16.27	8	0.000
	latitude + elevation	102.51	19.49	5	0.000
	host species + latitude + elevation	105.20	22.18	7	0.000
	host species*latitude + elevation	116.03	33.00	9	0.000
