# Supplementary material for: Drivers of community turnover differ between avian hemoparasite genera along a North American latitudinal gradient
Source: Ecol Evol. 2020 Jun 9;10(12):5402–15. doi: 10.1002/ece3.6283 (PMC7319150; doi:10.1002/ece3.6283)
Supplement: Supplementary file 1 — Appendix S1 [file ECE3-10-5402-s001.docx]

**Drivers of community turnover differ between avian haemoparasite genera along a North American latitudinal gradient.**

^1,2^* Naima C. Starkloff, ^2^ Jeremy J. Kirchman, ^3^ Andrew W. Jones, ^4^ Benjamin M. Winger, ^1^ Yen-Hua Huang, ^5,6^ Paulo C. Pulgarín-R, ^1^ Wendy C. Turner

**Appendix 1**: Comparison of linear mixed effects models predicting the prevalence and alpha diversity of *Leucocytozoon* and *Plasmodium* parasites in three closely related species of *Catharus* thrushes (n=414). We did not model *Haemoproteus* infection status as infections were rare (mean 7% infected). Models are ranked in ascending order of ∆AICc, relative to the model with the lowest AICc for that parasite genus (bolded). The lowest AICc is indicative of the best model in the set. K indicates the number of parameters in each model. W represents the Akaike weight which is a measure of probability of the model being the best in the set. All models included sampling timespan as random effects including the intercept only model. Prevalence was arcsine transformed and alpha diversity was quantified by extrapolating the Shannon diversity Index at each site.

| **Dependent variable** | **Variables included** | **AICc** | **ΔAICc** | **K** | **W** |
| --- | --- | --- | --- | --- | --- |
| ***Leucocytozoon* prevalence** | **intercept only** | **19.98** | **0.00** | **3** | **0.899** |
|  | host | 24.86 | 4.88 | 5 | 0.078 |
|  | host+latitude | 28.32 | 8.35 | 6 | 0.014 |
|  | latitude | 29.27 | 9.29 | 4 | 0.009 |
|  | elevation | 38.60 | 18.62 | 4 | 0.000 |
|  | host+elevation | 41.28 | 21.30 | 6 | 0.000 |
|  | host*latitude | 44.58 | 24.61 | 8 | 0.000 |
|  | latitude+elevation | 47.96 | 27.99 | 5 | 0.000 |
|  | host+latitude+elevation | 48.21 | 28.24 | 7 | 0.000 |
|  | host*latitude+elevation | 65.27 | 45.30 | 9 | 0.000 |
| ***Plasmodium* prevalence** | **intercept only** | **-8.26** | **0.00** | **3** | **0.934** |
|  | latitude | -2.72 | 5.54 | 4 | 0.058 |
|  | host | 1.29 | 9.55 | 5 | 0.008 |
|  | host+latitude | 10.66 | 18.92 | 6 | 0.000 |
|  | elevation | 11.83 | 20.09 | 4 | 0.000 |
|  | latitude+elevation | 17.38 | 25.64 | 5 | 0.000 |
|  | host+elevation | 21.85 | 30.11 | 6 | 0.000 |
|  | host*latitude | 29.38 | 37.63 | 8 | 0.000 |
|  | host+latitude+elevation | 31.65 | 39.90 | 7 | 0.000 |
|  | host*latitude+elevation | 52.01 | 60.27 | 9 | 0.000 |
| ***Leucocytozoon* diversity** | **intercept only** | **80.09** | **0.00** | **3** | **0.735** |
|  | host | 83.97 | 3.87 | 5 | 0.106 |
|  | latitude | 83.97 | 3.88 | 4 | 0.106 |
|  | host+latitude | 85.42 | 5.33 | 6 | 0.051 |
|  | host*latitude | 92.95 | 12.86 | 8 | 0.001 |
|  | elevation | 95.69 | 15.60 | 4 | 0.000 |
|  | host+latitude+elevation | 97.81 | 17.72 | 7 | 0.000 |
|  | latitude+elevation | 99.28 | 19.19 | 5 | 0.000 |
|  | host+elevation | 100.06 | 19.97 | 6 | 0.000 |
|  | host*latitude+elevation | 108.39 | 28.30 | 9 | 0.000 |
| ***Plasmodium* diversity** | **host** | **83.02** | **0.00** | **5** | **0.701** |
|  | intercept only | 85.09 | 2.07 | 3 | 0.249 |
|  | host+latitude | 89.29 | 6.26 | 6 | 0.031 |
|  | latitude | 90.28 | 7.25 | 4 | 0.019 |
|  | host+elevation | 99.04 | 16.02 | 6 | 0.000 |
|  | elevation | 99.19 | 16.16 | 4 | 0.000 |
|  | host*latitude | 99.29 | 16.27 | 8 | 0.000 |
|  | latitude+elevation | 102.51 | 19.49 | 5 | 0.000 |
|  | host+latitude+elevation | 105.20 | 22.18 | 7 | 0.000 |
|  | host*latitude+elevation | 116.03 | 33.00 | 9 | 0.000 |
